# Should topiramate be initial therapy in the management of idiopathic intracranial hypertension?: A literature review

**DOI:** 10.1097/MD.0000000000035545

**Published:** 2023-10-20

**Authors:** Abhishek Goyal, Katherine Zarroli

**Affiliations:** a Kasturba Medical College, MAHE, Manipal, India; b Department of Neurology, University of Florida College of Medicine - Jacksonville, Jacksonville, FL, USA.

**Keywords:** acetazolamide, idiopathic intracranial hypertension, topiramate

## Abstract

Idiopathic intracranial hypertension (IIH) is a condition of unknown etiology that primarily affects obese women of childbearing age. Symptoms include disabling headaches, visual disturbances, and intracranial noises (pulsatile tinnitus). Currently, no standardized treatment guidelines are available and the current management focuses on weight loss and acetazolamide use. There is an increasing body of evidence suggesting that the initial use of topiramate may be considered in IIH treatment. Acetazolamide is the recommended initial treatment for IIH, with topiramate often used as a second-line agent. Topiramate has multiple benefits to indicate it would pose effective in IIH management. Through varying mechanisms, it leads to weight loss and improves migraine headache control, the most common headache phenotype in IIH. Topiramate also inhibits the carbonic anhydrase enzyme like acetazolamide to reduce intracranial pressure and treat papilledema. The safety profile of topiramate is comparable or superior to acetazolamide. To date, there are limited studies comparing topiramate to acetazolamide or other treatment modalities in IIH. Based on its varying mechanisms of action, topiramate is a strong potential treatment agent for IIH, yet acetazolamide is often chosen first-line. However, the data supporting use of acetazolamide or topiramate is inefficient to designate one agent preferred over the other. There is a need for further studies assessing topiramate use in the treatment of IIH, and comparing topiramate use to other treatment modalities.

## 1. Introduction

Idiopathic intracranial hypertension (IIH) is a condition of unknown etiology that primarily affects obese women of childbearing age.^[1]^ The incidence is on the rise, mirroring that of obesity worldwide.^[2,3]^ Symptoms include disabling headaches, visual disturbances, and intracranial noises (pulsatile tinnitus). There are no standardized treatment guidelines; current management focuses on weight loss and acetazolamide use. In severe cases where progressive visual loss is present, surgical options can be considered including optic nerve sheath fenestration and cerebrospinal fluid (CSF) shunting.

Despite the suggestion that acetazolamide be an initial treatment for patients with IIH, a Cochrane review in 2015 concluded that there is insufficient evidence to recommend use of acetazolamide.^[4]^ There is an increasing body of evidence to suggest that the use of topiramate may be beneficial in the initial treatment of IIH. In this review, we explore the gaps in our current treatment recommendations and discuss the mechanisms by which topiramate may be uniquely effective in IIH treatment, suggesting it may be considered as an initial treatment.

## 2. Methods

A broad computer-aided review of the databases of published work (PubMed) was performed. The reference lists of included studies were screened and included as appropriate. Only studies available in English and in full text were reviewed. The literature search was performed between February 2023 and April 2023. The following keywords were used: IIH, pseudotumor cerebri, topiramate, and acetazolamide. A total of 29 articles were included for the purpose of this review. Our review included both basic science and clinical studies evaluating treatment modalities for IIH.

### 2.1. Diagnosis of idiopathic intracranial hypertension (IIH)

IIH was first recognized in 1893 after Quincke described a case of *serous meningitis.*^[5]^ The criteria for diagnosis was first defined by Dandy in 1937^[6]^ and later refined by Smith in 1985 to be known as the “Modified Dandy Criteria.”^[7]^ The most recent diagnostic criteria for definite IIH was proposed by Friedman and Jacobson in 2013 and requires: papilledema, a normal neurological examination (except for cranial nerve abnormalities), normal brain parenchyma without hydrocephalus, mass, or structural lesion and no abnormal meningeal enhancement or venous sinus thrombosis on MRI and MR venography, normal CSF composition, and elevated CSF opening pressure (≥250 mm CSF in adults) in a properly performed lumbar puncture.^[8]^

### 2.2. Current treatment guidelines

Standardized treatment options in IIH focus on weight loss and pharmacologic therapy with acetazolamide. This segment will focus on the data on the use of these treatment modalities for IIH.

### 2.3. Weight loss

Weight loss is recommended for all patients with obesity and IIH. In a prospective study including 22 participants, a nutritionally complete low calorie diet (425 Kcal/day) resulted in a significant decrease in intracranial pressure (ICP) of −8 cm H_2_O (SD4.2) from baseline 39.8 cm H_2_O (SD 5.1) with significant improvements in headache and papilledema.^[9]^ In patients with morbid obesity, bariatric surgical intervention may be an option for treatment as well. A randomized control trial of 66 participants comparing bariatric surgery and a community weight management in patients with body mass index (BMI) over 35 suggested bariatric surgery was significantly more effective in lowering ICP.^[10]^ Mean opening pressure decreased from 34.8 cm CSF (SD 5.8) at baseline to 26.4 cm CSF at 12 months (SD 8.7) in the surgery arm (*P* < .001). In the weight management arm, there was only a decrease from 34.6 (SD5.6) to 32 (SD 5.2); this was not statistically significant (*P* = .8). At both 12 and 24 months, improvement in weight, BMI, and reduction of excess body weight were significantly greater in the surgery arm versus the weight management arm (*P* < .001) with increased effect between 12 and 24 months.

### 2.4. Acetazolamide

Acetazolamide is currently the suggested first-line choice for pharmacological treatment of IIH.^[11]^ Acetazolamide acts as a carbonic anhydrase (CA) inhibitor to reduce ion and water transport across the choroid plexus and reduce CSF secretion. However, the data to support acetazolamide efficacy in IIH is lacking. To date, there are only 2 randomized controlled trials (RCTs) evaluating the use of acetazolamide in IIH.^[12,13]^ The first RCT by Ball et al (2011) found that acetazolamide was associated with an improvement in headache control (presence versus absence), visual loss, and transient visual obscurations. However, the study did not find evidence of reduction in papilledema, headache severity, improvement in visual acuity, or visual field outcomes. Furthermore, it was unblinded and underpowered with only 50 participants. Acetazolamide was allocated to 25 participants but only 13 completed the study.^[12]^

In the idiopathic intracranial hypertension treatment trial, use of acetazolamide and low sodium weight reduction diet compared with diet alone showed a modest improvement in visual field function.^[13]^ Vision loss was quantified by perimetric mean deviation (PMD) using Humphreys field analyzer. PMD in the acetazolamide group improved to −2.1 dB from a baseline of −3.53 dB whereas for the placebo group it improved to −2.82 dB. Although this was statistically significant (*P* < .05), the difference between the 2 groups was <1 dB. Furthermore, the clinical significance of this improvement was undetermined. Vision related quality of life questionnaires revealed an improvement with acetazolamide; however, they did not reach statistical significance. Additionally, there are some limitations to this study. It involved a specific subset of patients with narrow range of vision loss quantified by PMD of −2 to −7 dB. The role of acetazolamide in patients with asymptomatic papilledema is unclear. Furthermore, the most common presentation of IIH is headaches, over which acetazolamide showed no benefit over placebo. Headaches were assessed using headache impact test 6 questionnaire. There was no significant difference in improvement of headache impact test 6 score between acetazolamide (−9.56 ± 1.05) and placebo (−9.11 ± 1.14) at 6 months. A Cochrane review of the 2 RCTs in 2015 concluded that there is insufficient evidence to recommend acetazolamide as first line intervention for IIH.

### 2.5. Topiramate

Topiramate was originally formulated as an analogue of fructose 1,6 bisphosphate with the intention of inhibiting fructose 1,6 bisphosphatase and thereby blocking gluconeogenesis. It is a derivative of naturally occurring monosaccharide D-fructose and contains a sulfamate group, which resembles the sulfonamide group of acetazolamide.^[14]^ Additionally, it is a CA inhibitor, which can potentially reduce CSF secretion and decrease ICP much like acetazolamide.

Currently, topiramate is used as a second-line drug in the treatment of IIH based on its mechanism of action. It has an independent ability to induce weight loss, which is useful in obese females with IIH, and it has a strong efficacy in decreasing the frequency of headaches, the most common symptom of IIH.

### 2.6. Topiramate and weight loss

Weight loss is a known side effect of topiramate therapy. In a prospective open labeled study of 49 patients, patients baseline weights were reduced in 82% at 3 months and 86% at 1 year. Obese patients (BMI > 30 kg/m^2^) reported a higher weight loss versus mean (4.2 versus 3 kg at 3 months and 5.9 versus 10 kg at 1 year).^[15]^ In a meta-analysis of 10 studies, topiramate therapy of 96 to 200 mg a day was associated with an average weight loss of 6.58 kg for treatment durations of >28 weeks and 4.11 kgs for treatment durations of <28 weeks with minimum of 16 weeks of therapy.^[16]^

The exact mechanism by which topiramate leads to weight loss is unknown. A combination of decreased calorie intake, increased adiponectin levels, and improved leptin sensitivity have been proposed. Reduced caloric intake has been hypothesized to occur secondary to increased hypothalamic cortisol releasing hormone, which inhibits feeding behavior.^[17]^ Adiponectin is a protein hormone released by the adipose tissue. Elevated adiponectin levels have been shown to improve insulin sensitivity, which lead to increased uptake and utilization of glucose, and peripheral fatty acid oxidation.^[18]^ Leptin is another hormone released by the adipose tissue, which is known to induce satiety. Leptin levels may be elevated in obese patients due to leptin resistance. Topiramate induced weight loss decreases leptin levels and improves leptin sensitivity.^[19]^ In patients who lost 10% or more of their body weight during topiramate treatment, baseline leptin levels were reduced by 36 percent at 3 months and 16 percent at 1 year.^[14]^

Topiramate inhibition of CA could also affect lipogenesis in the mitochondria and cytosol leading to fat loss.^[20]^ The topiramate-phentermine combination pill is currently food and drug administration approved for the treatment of obesity. These mechanisms suggest that topiramate has an excellent effect in weight loss, which is a key recommendation for patients with IIH.

### 2.7. Topiramate and papilledema

As a CA inhibitor, topiramate may be effective in the management of papilledema and improve visual outcomes in patients with IIH. CA is a highly efficient enzyme which catalyzes the reaction between carbon dioxide and water to form bicarbonate and hydrogen ions which are critical to acid base equilibrium.^[21]^ In humans, there are 12 known isoforms of CA with CA II and XII being the dominant isoforms in the choroid plexus.^[22]^ A study determining the inhibition constant (K_f_) against their isoforms showed that acetazolamide had a higher K_f_ of 12 versus 10 for topiramate against CA II and 5.7 versus 3.8 for topiramate against CA XII. Lower K_f_ of topiramate corresponds to stronger affinity to CA isoform, suggesting topiramate could more potent than acetazolamide in reducing CSF secretion.^[21]^ In another study comparing the efficacy of topiramate versus acetazolamide against a control of saline in healthy rats, oral topiramate demonstrated a significant reduction in ICP by 22% versus 5% for acetazolamide.^[22]^ Furthermore, topiramate is more lipophilic than acetazolamide and hence would be able to cross the blood-brain barrier more easily to achieve a higher intracellular concentration.^[22]^ These findings suggest topiramate may be effective in the management of papilledema due to its mechanism of action, though studies need to be performed in humans to further analyze this hypothesis.

### 2.8. Topiramate and headache

Headache is the most common symptom of IIH. In the idiopathic intracranial hypertension treatment trial, approximately 84% of the participants had headaches. The phenotype of the headaches can vary considerably; however, migraine appeared to be most common, affecting approximately 52% of patients.^[23]^ In another prospective observational study, migraine was the predominant phenotype in 68% of patients with IIH.^[24]^ Other headache phenotypes less commonly observed include tension type and a combination of migraine and tension type headache.

Topiramate has multiple mechanisms by which it is effective in the treatment of migraine. It blocks voltage-gated sodium channels, inhibits kainate-type glutamate receptors, decreases L-type voltage-sensitive calcium currents, increases the opening of GABA-mediated chloride channels, inhibits CA, and increases the potassium conductance.^[25]^ Furthermore, topiramate has been shown to significantly inhibit calcitonin gene related peptide release in in ganglion cell cultures.^[26]^ All of these processes lead to reduced neuronal excitation and help prevent migraine headaches.

In a large randomized control trial of over 483 patients, topiramate at doses of 100 and 200 mg/day demonstrated statistically significant reduction in frequency of migraines.^[27]^ It is currently food and drug administration approved for the prophylaxis of migraine. Since migraine appears to be the predominant phenotype in IIH, topiramate can be effective for symptom control.

### 2.9. Side effects of topiramate

The safety profile of topiramate is comparable and possibly even superior to acetazolamide. Commonly reported adverse effects of topiramate therapy include paraesthesias, fatigue, nausea, anorexia, dizziness, diarrhea, weight loss, difficulty with concentration/attention, and somnolence.^[28]^ There is scant data for side effects of topiramate in IIH treatment. However, there are many studies reviewing its safety profile in epilepsy and migraine prophylaxis.

An analysis of safety and tolerability data from 1580 patients receiving topiramate for migraine prevention in controlled trials concluded topiramate is generally safe and well tolerated. Adverse events leading to dose reduction or interruption of treatment occurred in 15%, 20%, and 24% of patients in the topiramate 50, 100, 200 mg/day groups. These side effects more frequently occurred in the initial dose titration phase than the maintenance phase.^[28]^ A meta-analysis of 10 studies reviewing topiramate in the treatment of obesity reported paraesthesia as the most common adverse effect. Approximately 446 participants out of 2628 (16.9%) had adverse effects leading to treatment withdrawal.^[16]^

In the RCTs by Ball AK et al and the IIH treatment trial evaluating use of acetazolamide in IIH, treatment discontinuation rates were 48% and 19% respectively.^[12,13]^ Common side effects were paraesthesia, dysgeusia, vomiting, and diarrhea.

## 3. Conclusion

Topiramate is a well-known drug most frequently used as an anti-seizure medication, migraine preventative, and/or weight loss agent. There is a growing body of literature to suggest that topiramate may be a useful in the initial management of IIH.

To date, there is only 1 study that has compared topiramate and acetazolamide in the treatment of IIH. It concluded that there is no statistically significant difference between the 2 medications, though topiramate reported prominent weight loss. This study had multiple shortcomings. It was a single center open label study with a small sample size of 40. There was also no placebo arm in the study.^[29]^ There have been no other studies comparing topiramate to other treatment modalities, such as weight loss or bariatric surgery.

Currently, acetazolamide remains the preferred the initial pharmacotherapy for IIH treatment. However, a Cochrane review in 2015, which included the 2 RCTs, concluded that the evidence to recommend its use is inadequate.^[4]^ Topiramate is an excellent alternative for use in IIH. It has multiple beneficial mechanisms including weight loss, decreased CSF secretion, and reduction in the occurrence of headaches. Notably, the limitations to the use of topiramate include the unknown optimal dose and limited data on efficacy, based only on open label studies, a few case reports, and animal studies. At present, the use of topiramate in clinical practice is limited to patients with significant headaches with acetazolamide typically preferred by clinicians due to its predictable effect and familiarity. Our review highlights the need for future studies evaluating the use of topiramate in IIH, as topiramate in theory may pose particularly effective as an initial therapy. This is important as, ultimately, findings could improve patient healthcare outcomes and quality of life.

## Author contributions

**Conceptualization:** Abhishek Goyal, Katherine Zarroli.

**Formal analysis:** Abhishek Goyal.

**Investigation:** Abhishek Goyal.

**Methodology:** Abhishek Goyal.

**Supervision:** Katherine Zarroli.

**Writing – original draft:** Abhishek Goyal, Katherine Zarroli.

**Writing – review & editing:** Abhishek Goyal, Katherine Zarroli.
